# Virtual Clinical Trials Guided Design of an Age-Appropriate Formulation and Dosing Strategy of Nifedipine for Paediatric Use

**DOI:** 10.3390/pharmaceutics15020556

**Published:** 2023-02-07

**Authors:** Dilawar Khan, Raj Badhan, Daniel J. Kirby, Simon Bryson, Maryam Shah, Afzal Rahman Mohammed

**Affiliations:** 1 Aston Pharmacy School, College of Health and Life Sciences, Aston University, Birmingham B4 7ET, UK; 2 Proveca Ltd., No. 1 Spinningfields, Quay Street, Manchester M3 3JE, UK

**Keywords:** paediatrics, PBPK, pharmacokinetics, age-appropriate, mini-tablets, modified release, adherence

## Abstract

The rapid onset of action of nifedipine causes a precipitous reduction in blood pressure leading to adverse effects associated with reflex sympathetic nervous system (SNS) activation, including tachycardia and worsening myocardial and cerebrovascular ischemia. As a result, short acting nifedipine preparations are not recommended. However, importantly, there are no modified release preparations of nifedipine authorised for paediatric use, and hence a paucity of clinical studies reporting pharmacokinetics data in paediatrics. Pharmacokinetic parameters may differ significantly between children and adults due to anatomical and physiological differences, often resulting in sub therapeutic and/or toxic plasma concentrations of medication. However, in the field of paediatric pharmacokinetics, the use of pharmacokinetic modelling, particularly physiological-based pharmacokinetics (PBPK), has revolutionised the ability to extrapolate drug pharmacokinetics across age groups, allowing for pragmatic determination of paediatric plasma concentrations to support drug licensing and clinical dosing. In order to pragmatically assess the translation of resultant dissolution profiles to the paediatric populations, virtual clinical trials simulations were conducted. In the context of formulation development, the use of PBPK modelling allowed the determination of optimised formulations that achieved plasma concentrations within the target therapeutic window throughout the dosing strategy. A 5 mg sustained release mini-tablet was successfully developed with the duration of release extending over 24 h and an informed optimised dosing strategy of 450 µg/kg twice daily. The resulting formulation provides flexible dosing opportunities, improves patient adherence by reducing frequent administration burden and enhances patient safety profiles by maintaining efficacious levels of consistent drug plasma levels over a sustained period of time.

## 1. Introduction

Nifedipine is a dihydropyridine calcium channel blocker that exerts its effect directly on vascular smooth muscle and myocardial cells and inhibits the influx of calcium ions by blocking voltage dependent L-type Ca^2+^ channels. This results in reduced intracellular calcium, thereby reducing peripheral arterial vascular resistance and dilatation of coronary arteries, leading to improved myocardial oxygen delivery and reduced blood pressure [1]. Nifedipine is rapidly and completely absorbed from the gastrointestinal tract. The oral bioavailability of nifedipine is approximately 70%, with peak plasma levels attained within 30 to 45 min [2]. Nifedipine is around 95% protein bound and has an elimination half-life of about 2 to 5 h [2].

The use of nifedipine was once well established, being one of the most widely prescribed medicines to treat hypertension. However, owing to safety and tolerability concerns and with the introduction of newer agents, the use of nifedipine has become less desirable [3]. In 1995, concerns regarding the safety of nifedipine surfaced after a meta-analysis of clinical trials of nifedipine was published. The report concluded that the use of short-acting (SA) nifedipine in moderate to high doses resulted in an increase in total mortality in patients with coronary heart disease [4]. Other reports have also mentioned safety concerns with SA nifedipine preparations, associating its use with cerebral ischemia and myocardial infarction [5,6]. As opposed to concerns regarding the safety of SA nifedipine in adults, studies evaluating the safety of SA nifedipine in children suggest otherwise, with several studies concluding SA nifedipine to be an important, safe and effective oral antihypertensive agent [7,8,9].

The advantages of SA nifedipine use are the rapid onset of action and lack of central nervous system (CNS) depression; however, the precipitous reduction in blood pressure means nifedipine is associated with adverse effects, including reflex tachycardia, retinal ischemia and myocardial ischemia and infarction [10]. Consequently, long-acting formulations have become available, addressing the drawbacks of SA nifedipine preparations. However, importantly, there are no modified release preparations of nifedipine authorised for paediatric use, and hence a paucity of clinical studies reporting pharmacokinetics data in paediatrics. Owing to a lack of appropriate formulations available, children are often given unlicensed preparations where current marketed dosage forms are manipulated prior to administration, increasing the risk of potential unknown adverse effects [11,12,13]. The challenge to develop such sophisticated dosage forms is due to limited formulation development strategies for such formulations alongside little to no work reported on modified/sustained release paediatric formulations. Challenges are further compounded by the distinct differences seen in the way in which drugs perform in the paediatric population. Further, performing clinical studies in children is a challenge as children are an exceptional population with specific ethical and clinical concerns. As a consequence of anatomical and physiological variances observed between children and adults, pharmacokinetic parameters can significantly differ and often lead to sub therapeutic and/or toxic plasma drug concentrations [14].

However, in the field of paediatric pharmacokinetics, the use of pharmacokinetic modelling, particularly physiological-based pharmacokinetics (PBPK), has revolutionised the ability to extrapolate drug pharmacokinetics across age groups, allowing for pragmatic determination of paediatric plasma concentrations to support drug licensing and clinical dosing. This approach relies on the established principles of mechanistic PBPK modelling to describe tissue volumes, tissue perfusion, renal/liver function, blood biochemistry and drug metabolism enzyme/and drug transporter protein expression [15], in addition to the inclusion of development changes [16,17,18,19,20,21,22].

Furthermore, PBPK modelling in paediatrics has gained deregulatory approval by the FDA to support new indications [23,24].

A range of software companies offers PBPK modelling platforms [25], including paediatrics, of which the Simcyp™ Simulator is widely used to predict drug performance from virtual population groups, including paediatrics [26,27], pregnancy [28,29,30] and specialised disease states such as oncology [31,32].

For the first time, an attempt to integrate PBPK modelling to a paediatric specific formulation development approach to clinically inform an appropriate dosage form design and strategy of nifedipine is made. An integrated approach such as this not only aims to inform dosage form design to improve formulation safety profiles but also to provide the necessary paradigm shift in paediatric formulation development that is vital for avoiding unnecessary paediatric studies, ensuring clinical trial dose selection is based on science and for minimising clinical trial enrolment.

In the present study, several grades and concentrations of HPMC were compared for their effect on release rate. The resulting dissolution profiles were then evaluated to determine the mechanism of release using kinetic modelling. In order to pragmatically assess the translation of resultant dissolution profiles to the paediatric populations, virtual clinical trials simulations were conducted. In the context of formulation development, the use of PBPK modelling allowed the determination of optimised formulations that achieved plasma concentrations within the target therapeutic window throughout the dosing strategy.

## 2. Materials and Methods

### 2.1. Materials

Nifedipine was purchased from Alfa Aesar (Lancashire, UK). Lactose monohydrate and magnesium stearate were obtained from Sigma-Aldrich (Dorset, UK), whilst microcrystalline cellulose (MCC) as Pharmacel 102 was obtained from DFE Pharma (Goch, Germany). Colloidal silica dioxide (Aerosil 200) was obtained from Evonik Industries (Essan, Germany) and grades of hydroxypropyl methylcellulose (HPMC) as METHOCEL™ were obtained from Colorcon (Dartford Kent, UK). Market extended release nifedipine preparations including Nifedipress MR 10 and Adalat LA 30 were obtained from Dexcel Pharma (Daventry, UK) and Bayer (Reading, UK).

Dissolution media comprised of a phosphate/citrate buffer with Sodium lauryl sulphate (SLS). Dibasic sodium phosphate and citric acid were purchased from Acros Organics (Morris Plains, NJ, USA), whereas phosphoric acid and SLS were obtained from Sigma-Aldrich (Dorset, UK).

For sample analysis using HPLC: Trifluoroacetic acid (TFA) for HPLC (≥99.0%), was obtained from Sigma-Aldrich (Dorset, UK), whilst acetonitrile and methanol were purchased from Fisher Scientific (London, UK).

### 2.2. HPLC Analytical Method Development

Sample analysis was carried out using an Agilent 1260 series (Agilent Technologies, Santa Clara, CA, USA), using a reverse-phased Eclipse plus C18, 4.6 × 150 mm, 3.5 μm column (Agilent Technologies, Santa Clara, CA, USA). 

Separation of nifedipine was achieved using an isocratic mobile phase comprised of TFA:ACN (25:75 *v*/*v*). The flow rate was set at 0.8 mL/min and a wavelength of 235 nm was used for detection. Calibration curve ranging from 0.39 to 100 µg/mL (9-point) was generated by serial dilution (1:2 dilution) in methanol. Samples prepared for analysis were diluted to fall within the calibration range. Method validation was performed according to International Council for Harmonization (ICH) (Q2 (R1)) guidelines.

### 2.3. Dose Banding and Selection of Strengths

The dose of nifedipine indicated for the treatment of hypertension in children between the ages of 1 month and 11 years is 200–300 μg/kg 3 times a day. The frequency of dosing depends on the preparation used; however, there is no standardised modified preparation nifedipine available for paediatric use. The selection of strengths was based on dosing requirements according to the BNF and reported optimised dose strategy post PBPK modelling.

### 2.4. Dissolution

Paediatric specific biorelevant media was not employed owing to previous findings where nifedipine did not show any significant age-related difference in dissolution of nifedipine when compared to adult biorelevant media [33]. Similarly, evaluation of the effect of age-related difference on dissolution on another BCS class II drug (carvedilol) did not show any significant effect of varying physiological differences on drug release profiles [34]. Therefore, dissolution media was prepared as per the USP monograph for nifedipine extended-release tablets. In brief, 330.9 g of dibasic sodium phosphate and 38 g of citric acid were dissolved in water in a 1 L volumetric flask. 10 mL of phosphoric acid was then added, and the resulting concentrate was diluted with water to volume. 125.0 mL of concentrate (buffer) and 1 L of 10% sodium lauryl sulphate solution were mixed and diluted to 10 L. The medium was adjusted to a pH of 6.8.

All dissolution tests were carried out using an Erweka DT 126 with USP 2 paddle apparatus (Langen, Germany). Each vessel contained 900 mL of media, maintained at a temperature of 37 °C with a continuous paddle speed of 50 rpm. 5 mL samples were drawn at identified time points (2, 3, 4, 5, 6, 8, 12, 20 and 24 h) and replaced with 5 mL of fresh media in order to maintain sink conditions. Drug release was quantified using HPLC and adjusted for cumulative release (%). Data presented as mean ± standard deviation (*n* = 6).

### 2.5. Kinetic Modelling

In an effort to further understand and compare the mechanism of nifedipine release from formulations, dissolution data were fitted within the following four kinetic models: zero order, first order, Higuchi and Korsmeyer–Peppas models. Method for data fitting was followed as described by Costa, P. and J. M. Sousa Lobo (2001) [35] (Table 1).

### 2.6. Extended-Release Mini Tablet Production

Mini-tablets were processed using a Specac semi-automatic hydraulic press (Slough, UK) equipped with 4 mm multiple (three) tipped concave punches at a target drug loading of 10% *w/w* and a tablet mass of 50 mg. Tablets were compressed using a compression force of 10 kN with quick release. Optimised blending and tabletting parameters for low dose mini-tablets were applied as described by Khan, D., et al. (2021) [34].

### 2.7. Hardness

Tablet hardness was evaluated using a Copley TBF 100 hardness tester (Nottingham, UK) which measures the force required to break the tablet. Hardness values were measured in Newtons. Data presented as mean ± standard deviation (*n* = 3).

### 2.8. Friability

Friability testing was performed using a Sotax F2 Friabilator (Allschwill, Switzerland) to measure the ability of the mini-tablets to resist mechanical stress. Ten tablets were lightly brushed with a soft brush and an initial weight was determined. The tablets were then placed in a rotating drum and spun at a speed of 25 rpm for 4 min (100 revolutions total). The tablets were removed, dusted and the final weight was determined. Percent friability was calculated using the following formula:

% Friability = (initial weight − final weight)/initial weight × 100

### 2.9. Development of a Nifedipine PBPK Model for Use in Virtual Clinical Trials

In order to pragmatically assess the translation of resultant dissolution profiles to the paediatric populations, virtual clinical trials simulations were conducted using the physiologically-based pharmacokinetic (PBPK) modelling tool Simcyp in both adults and children (Simcyp Ltd., a Certara company, Sheffield, UK, Version 21). Unless otherwise stated, mixed genders (50:50) were incorporated into all simulations. In addition, all observed nifedipine concentrations used for the PBPK modelling in both adult and paediatric populations were extracted from publicly available data. Nevertheless, predicted concentrations were generated from the manufactured novel extended release mini tablets based on the dissolution profiles (Section 3.5).

#### 2.9.1. Validation in Adults

We utilised the previously validated and published nifedipine PBPK model incorporated [36] into the Simcyp Simulator (See Supplementary Materials Table S1), as the basis for downstream extrapolation to paediatrics. We considered 4 studies within healthy adult populations: (i) 18 healthy male volunteers (23–29 years old) received a single oral dose of nifedipine 20 mg (immediate release) in 3 studies [37]; (ii) 6 healthy male volunteers (aged 20–25 years old) received a single oral dose of nifedipine 20 mg (immediate release) [38]; (iii) 6 healthy male volunteers (aged 20–30 years old) received a single oral dose of nifedipine 20 mg (immediate release) [39]; (iv) 6 healthy male volunteers (aged 22–34 years old) received nifedipine 10 mg (immediate release) as a single dose or three times a day for 5 days [40].

In order to simulate an MR formulation system, a Weibull function [41] (Equation (1)) was fit to a 30 mg modified release reference in vitro release profile (Adalat OROS^®^) [42]:

(1) $$\frac{M_{t}}{M_{\infty }}=1-e^{=at^{b}}$$

where *M_t_* is the accumulated mass dissolved at time *t*, *M_∞_* is the mass dissolved at infinite time, with *a* and *b* being the scale parameter and shape parameter, respectively.

The dissolution profile was then used within the Simcyp Simulator to model dissolution from the MR formulation, and compared to a study where 12 healthy adults were dosed 30 mg Adalat OROS^®^ as a single dose [42].

#### 2.9.2. Formulation Performance in Virtual Adults

In order to assess the performance of the prepared formulations in adults, the dissolution profiles for F1 (no HPMC), F2 (30% HPMC) and F3 (50% HPMC) were incorporated into a virtual trial (−10 × 10 trial design *n* = 100) with healthy adults aged 20–50 years of age with dosing at either 30 mg twice daily or 60 mg once daily for 6 days. The impact of HPMC content on pharmacokinetics were subsequently assessed, in the context of maintenance of plasma concentrations within the purposed therapeutic range (25–100 ng/mL) [43].

#### 2.9.3. Formulation Performance in Virtual Paediatrics

In order to examine the performance of formulations within virtual paediatrics, we utilised the Simcyp Paediatric model. This model implements the same generic whole-body PBPK model implemented within the adult model, but considers ontogeny throughout the paediatric age range, through defined physiological (including gut) and biochemical ontogeny functions [44].

For paediatric studies, the dissolution profiles for F1 (no HPMC), F2 (30% HPMC) and F3 (50% HPMC) were incorporated into a 10 × 10 trial design (*n* = 100) with children aged 5–11 years of age with dosing at 250 µg/kg once, twice or three times daily. Results were demarked for 5–7-year-olds and 7–11-year-olds. The impact of HPMC content on pharmacokinetics were subsequently assessed, in the context of maintenance of plasma concentrations within the purposed therapeutic range (25–100 ng/mL) [43].

#### 2.9.4. Developing a Dosing Approach in Children

Given the paucity of distinct nifedipine clinical studies reporting pharmacochemical data within paediatrics, in order to identify a possible dosing approach for use in children, dose adjustments were considered through 100 µg/kg increments to achieve the majority of subjects with trough plasma concentrations within the therapeutic window. The formulation attaining plasma concentration throughout the therapeutic window was selected and an identical trial design was utilised as described in Section 2.9.3.

#### 2.9.5. Predictive Performance

To confirm the predictive performance during validation, prediction of pharmacokinetic metrics to within two-fold (0.5–2.0 fold ratio) of that published in clinical data was accepted [45,46,47]. Furthermore, a visual predictive checking (VPC) strategy was utilised to visually compare predicted concentration–time profiles with retrospective observed data, with predictions valid when the predicted data points overlapped with the observed data sets [39,48,49]. Observed data were acquired from retrospective published studies, extracted using WebPlotDigitizer v.3.10 (http://arohatgi.info/WebPlotDigitizer/ (accessed on 15 September 2022)).

### 2.10. Statistical Analysis

Statistical analysis of the drug release profiles was carried out by one-way analysis of variance (ANOVA) with a post hoc Tukey test using GraphPad Prism version 8 (GraphPad Software, San Diego, CA, USA).

## 3. Results and Discussion

### 3.1. Acceptability of Mini-Tablets

Children are not small adults, nor are all children alike. Similarly, paediatric drug development is not a one-size-fits-all process. It relies on factors such as disease type, inherent physicochemical characteristics, age and maturity of the patient, and formulations/dosing options. Developing an age-appropriate formulation, especially for children, is an immense challenge from a scientific, ethical and logistical standpoint. This is due to a lack of translatable dosage form development technologies and the absence of paediatric clinical data. The use of PBPK aims to minimise drug related adverse effects and to inform formulation design and development, alongside contributing to the need of availability of more pharmacokinetic data in paediatrics.

In a recently published review article, an attempt to survey currently available evidence on the acceptability of oral medicine in paediatrics to guide the selection of appropriate dosage form types in future paediatric formulation development was made, resulting in several key findings, including the superior acceptability of small innovative dosage forms over other conventional dosage form types [50]. A particular study carried out by van Riet-Nales et al. (2013) evaluated the acceptability of a 4 mm mini tablet in 183 children aged 1–4 years. The study concluded that a 4 mm tablet is well accepted for children from the age of 12 months [51]. However, many of these studies reported that the children either chewed or crushed the tablets before swallowing. Therefore, the target age range for the novel 4 mm extended release mini tablets was specified from the age of 3 years and onwards, where the tablet is expected to be administered as a whole and therefore allowed to work in its intended extended fashion. Regarding children under the age of 3 years, we would recommended alternative dosage form types such as extended release granules or pellets; however, this is not within the scope of this study.

Furthermore, the appraisal of Paediatric Investigation Plans (PIPs) by regulators including the European Medicines Agency (EMA) and its Paediatric Committee (PDCO) demonstrates concurrence with the acceptability of small (0–4 mm) tablets in children aged 2–5 years of age [52]. Older children can either take multiple mini tablets or a conventional adult marketed modified release preparation.

### 3.2. Paediatric Formulation Development Approach for Extended Release Mini-Tablets and Formulation Composition Optimisation

When developing a paediatric specific formulation, the inclusion of excipients must be justified. There are several techniques to achieve modified release (MR) including matrix, membrane controlled and osmotic pump systems. In order to guide the selection of safe and age-appropriate excipients to achieve an extended release preparation, the compositions of current market nifedipine preparations were initially explored. Reference to the Safety and Toxicity of Excipients for Paediatrics (STEP) database was made to ensure the selection and load of excipients were paediatric compliant. However, upon evaluating, we found that the formulations were composed of numerous excipients (10 to 15). This not only added to cost but increased the potential of unknown excipient-related toxicity and safety concerns.

Moving forward, current modified release mini tablet preparations with a paediatric license were evaluated. As of now, there are only three modified release mini tablet preparations (Orfiril Long^®^, Slenyto and Pancrease MT^®^) licensed for paediatric use. However, the MR technique within these formulations use methacrylic acid copolymer, which has been associated with fibrosing colonopathy [53]. For such reasons, and to limit the total number of excipients within the formulation, it was decided to explore hydrophilic monolithic systems where HPMC is used as the gold standard. A monolithic system exposes all of its surface area for potential solubilisation and is a simple and effective technique where different grades of HPMC can be compared to ascertain a required baseline behaviour of disintegration and drug release. HPMC is hydrophilic, non-ionic, enzyme resistant, GRAS listed and appropriate for paediatric use at levels below 660–900 mg/kg per day [54]. Several grades of HMPC were explored to optimise drug release in order to achieve an administration of either once daily or twice daily.

Since low-viscosity HPMC is advised for low aqueous soluble substances such as nifedipine, we decided to firstly explore various concentrations of HPMC E3. In order to determine the baseline behaviour of disintegration and drug release with HPMC E3, three very different concentrations were used (0, 50 and 89.5% *w/w*). It was envisioned that since nifedipine is poorly soluble, HPMC E3 would provide erosion based extended release. However, all three formulations presented a similar drug release profile, suggesting that the effect of HPMC E3 on providing extended release was nominal. This is due to the low viscosity of this particular HPMC grade where the viscosity is 3 cP (of a 2% solution at 25 °C) [55].

Another observation made here was that total nifedipine release was limited to around 60%. This was due to the degradation of nifedipine where nifedipine is known to be highly sensitive to light. Upon exposure to light, nifedipine degrades to nitro- and nitroso-pyridine analogues by intramolecular processes, as well as a few minor secondary products generated from inter-molecular interactions between primary degradation products and their intermediates [56]. Moving forward, all dissolution experiments were conducted after the apparatus was fully covered to prevent any light entering the dissolution vessels.

**Figure 1:**
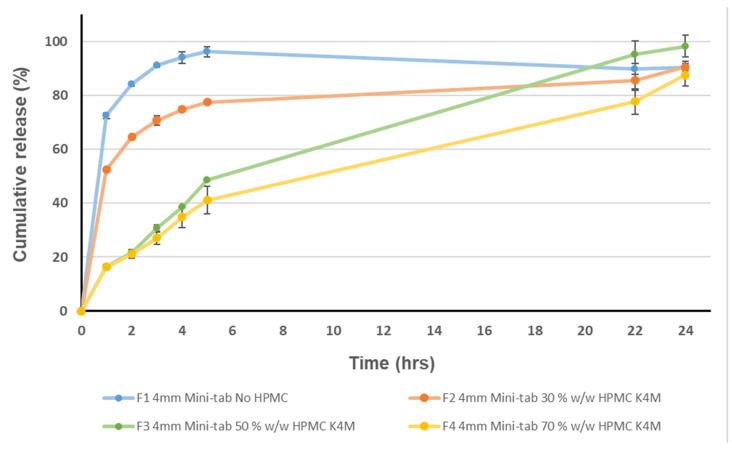
Dissolution profiles of mini-tablets composed of various grades of HPMC K4M. Data presented as mean ± standard deviation.

For an effective controlled-release matrix, rapid hydration is required to form a protective gelatinous layer, as it prevents the drug and excipients in the matrix from dissolving prematurely [57]. HPMC E3 not only displayed a slow rate of hydration but also formed a relatively weak gel layer. This was observed during the 6 h dissolution mark, where the tablet fully fragmented. The low viscosity of HPMC E3 suggests reduced ether groups were available for hydrogen bonding with water molecules, while the presence of poorly water soluble nifedipine resulted in further disruption of hydrogen bonding, thereby decreasing the amount of water bound to the polymer [58]. For these reasons, HPMC E3 was discounted and various concentrations of medium viscosity HPMC K4M were explored (Figure 1).

In general, as polymer viscosity increases, drug diffusion and release rates decrease. This is because high viscosity hypromellose results in a turbid gel that resists erosion and dilution, since hypromellose chains swell more quickly and prevents further liquid from entering the pores [57]. It follows that if a good gel layer is formed, the rate of drug release will be reduced and will be dependent on the rate at which drug molecules diffuse through it, as well as the rate of mechanical destruction of the gel layer by the attrition and unravelling of the matrix [57]. The effect of increasing concentrations of HPMC K4M on nifedipine release rate was significant (*p*-value < 0.05), as after 6 h the total drug released for F1 was 96.18 ± 1.76% and 77.43 ± 0.77%, 44.83 ± 0.33% and 41.11 ± 5.18% for F2, F3 and F4, respectively.

After comparing different concentrations of HPMC K4M, F3 was chosen as the optimum formulation that provided a release profile extending over 24 h (Figure 1). Other formulation constituents included lactose as the diluent and magnesium stearate and AEROSIL^®^ as tabletting excipients (Table 2). Lactose is considered to be safe because of its presence in various forms of milk, including breast milk and formula milk. Lactose is approved as an ‘additive’ by the FDA and listed as GRAS and has a recommended threshold of 5 g per dose [59].

The mixing and tabletting strategy employed was as described by Khan et al. (2021) [34], where optimised blending and processing parameters (mixing for 5 min at a speed of 250 rpm and compression force of 10 kN with quick release) resulted in mini-tablets that displayed good mechanical strength (62.10 ± 1.90 N and 0.25% friability) and content uniformity (98.57 ± 4.26%) that met pharmacopeia requirements.

Fitting the release data to the following models was used to study the kinetics of nifedipine release: zero order, first order, Higuchi and Korsmeyer–Peppas models (Table 3). Based on the correlation coefficient (R^2^) values, formulations containing HPMC most closely fit the Higuchi and Korsmeyer–Peppas kinetic model. The release exponent (*n*) was found to be 0.15 for F2 (suggesting a drug release mechanism by quasi-Fickian diffusion) and between 0.5 < *n* < 1 for F3 and F4 (anomalous (non-Fickian transport) drug transport mechanism) [60]. Nifedipine release in F1 (immediate release) followed first order kinetics; this was expected as the formulation did not possess any extended release properties. It is also important to mention that the release of nifedipine formulations containing HPMC displayed an initial burst release, followed by a decrease in release rate over time (Figure 1). This may have been due to the drug close to the matrix surface being released before the surrounding polymer reached the polymer disentanglement concentration, since the diffusion coefficients for drug molecules were higher than the polymer in the early stages [61]. Lastly, it is evident that the release rate of nifedipine from formulations containing HPMC K4M decreased as a function of increasing polymer concentration. This can be seen as a result of increasing linearity when release data were fitted to the Higuchi and Korsmeyer–Peppas kinetic model (Table 3).

### 3.3. Virtual Clinical Trials Analysis of Nifedipine Model in Adults and Children

Having identified optimal formulations and resulting dissolution profiles, we next attempted pragmatically to assess the in vivo translation using virtual clinical trials analysis.

These approaches utilised physiologically based pharmacokinetic modelling and knowledge of population variability in physiological and biochemical properties governing drugs absorption, distribution, metabolism, and elimination to develop virtual populations containing the matching inherent physiological and biochemical variability identified within clinical trials populations.

**Figure 2:**
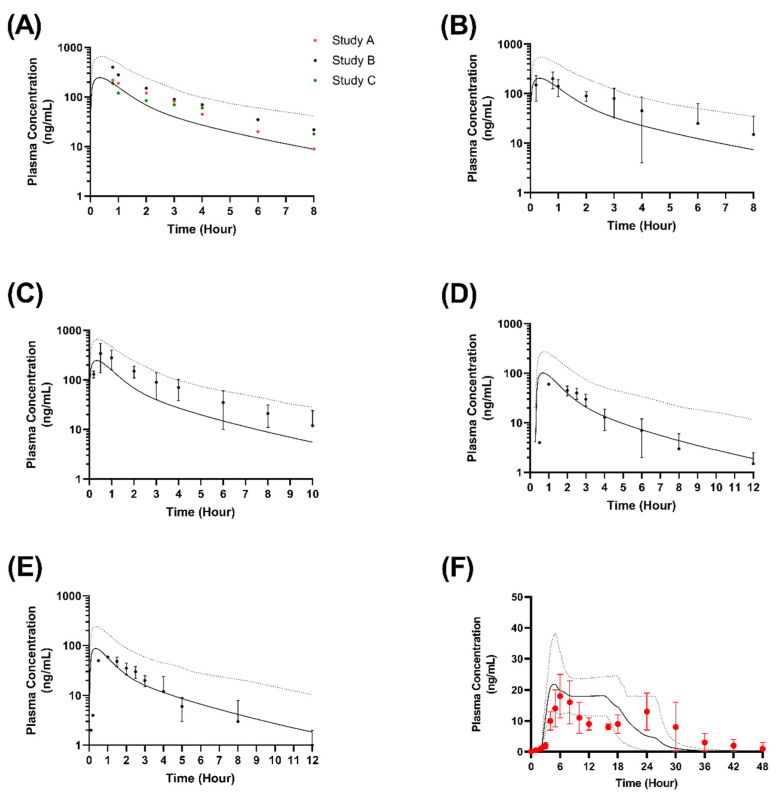
Simulated plasma concentration-time profiles of nifedipine in healthy adults. (**A**) Single oral dose of nifedipine 20 mg (immediate release) in 3 studies [37]; (**B**) Single oral dose of nifedipine 20 mg (immediate release) [38]; (**C**) Single oral dose of nifedipine 20 mg (immediate release) [39]; Nifedipine 10 mg (immediate release) as a single dose (**D**) [40] or three times a day for 5 days (**E**) [40]; (**F**) Single or dose of 30 mg modified release nifedipine [42]. Filled circles indicate the observed clinical data, with dotted lines indicating the corresponding 5th and 95th percentile range of the predicted mean (solid lines). Vertical lines indicate standard deviation.

In order to validate the applicability of the model in adults, we assessed the ability of the model to recapitulate retrospective nifedipine plasma concentration-time profiles in adults from four studies. Pharmacokinetic parameters were consistent with observed data and ranges (Figure 2), and within two-fold for the geometric mean of the reported pharmacokinetic parameters (Table 4), confirming successful validation. It would be important to mention that the variability in plasma concentration for the study carried out by Wonnerman et al. (2008) (Figure 2F) was due to the marked pH dependency of the test product, and because the study evaluated the pharmacokinetic properties when administered after a high-fat meal.

### 3.4. Formulation Performance in Virtual Adults

**Figure 3:**
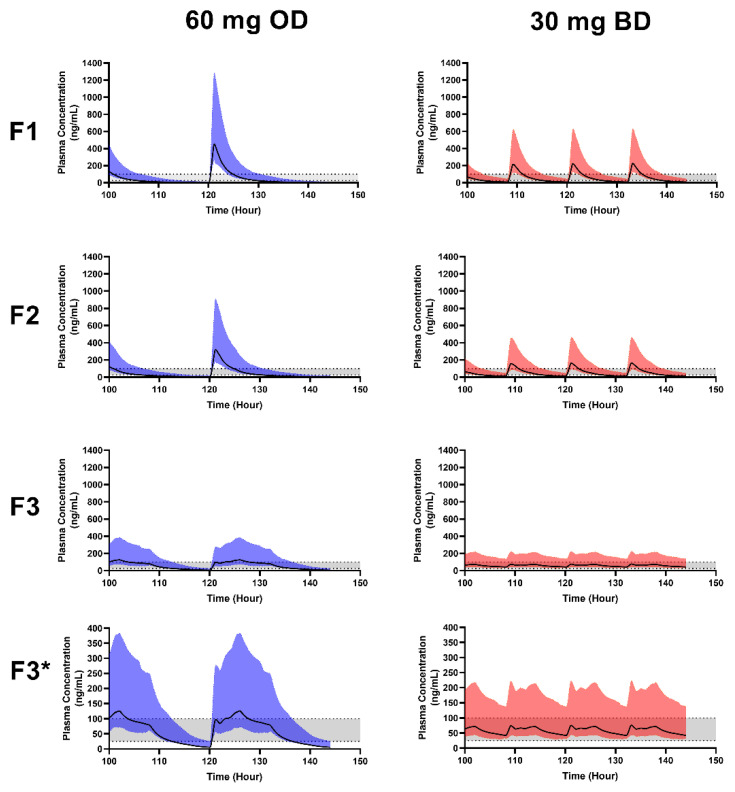
Simulated plasma concentration-time profiles of formulated nifedipine mini-tablets in healthy adults. The predicted plasma concentrations of nifedipine when formulated into F1 (no HPMC), F2 (30% HPMC) and F3 (50% HPMC) mini-tablets, following dosing at 60 mg once daily (left panels) or 30 mg twice daily (right panels). F3*: Represents F3 with an axis range from 0 to 400 ng/mL. Solid lines indicate predicted plasma concentrations, shaded regions indicate the 5th–95th percentile range around the predicted mean. The suggested therapeutic window (25–100 ng/mL) is indicated by the shaded horizontal region.

In order to pragmatically assess the impact of HPMC content on pharmacokinetics, dissolution profiles from formulations F1 (no HPMC), F2 (30% HPMC) and F3 (50% HPMC) mini-tablets were used to drive predictions of oral pharmacokinetics using the validated adult model. A dosage of 60 mg once daily and 30 mg twice daily was examined to identify an appropriate dosing approach to target the therapeutic range of 25–100 ng/mL [43] (Figure 3).

Formulation F3 provided a broadly lower Cmax for both twice and once daily dosing, 76.68 ± 39.5 ng/mL and 126.8 ± 62 ng/mL, respectively, compared to F1 and F2 (Table 5), with twice daily dosing mean plasma concentrations more appropriately targeting the therapeutic window throughout the dosing approach (Figure 3). The results from adults demonstrate the ability of the F3 mini-tablet to provide the required plasma concentration within the therapeutic window for a longer duration than that from F1 and F2, which provided a more rapid dissolution (as a result of the reduced HPMC content) and hence rapid absorption into the systemic circulation [62].

### 3.5. Formulation Performance in Virtual Paediatrics

In order to pragmatically assess the impact of HPMC content on pharmacokinetics in children, F1–F3 dissolution profiles were used to drive predictions of oral pharmacokinetics in children aged 5–7 and 7–11 years at a fixed dose of 250 µg/kg. A dose of 250 µg/kg was chosen as a median dose to the indicated dose of 200–300 μg/kg three times a day for children from aged 1 month to 11 years [63].

**Figure 4:**
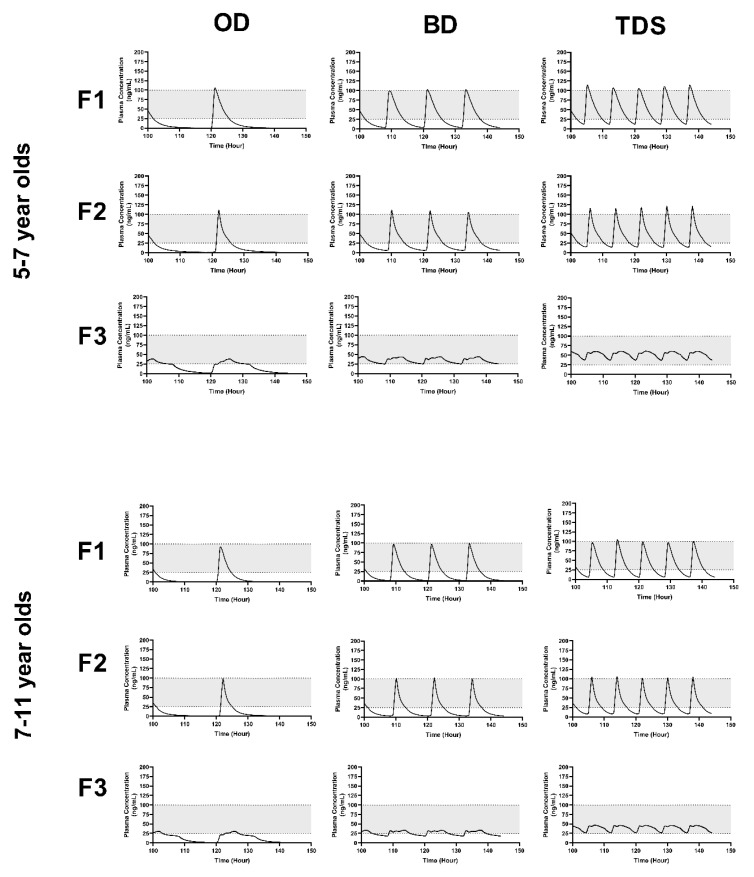
Simulated plasma concentration-time profiles of formulated nifedipine mini-tablets in children. The predicted plasma concentrations of nifedipine when formulated into F1 (no HPMC), F2 (30% HPMC) and F3 (50% HPMC) mini-tablets, following dosing at 250 µg/kg in children aged 5–7 years (top panels) or 7–11 years (bottom panels), following a once (OD), twice (BD) or three (TDS) times daily dosing (left, middle and right panels, respectively). Solid lines indicate predicted plasma concentrations, shaded regions indicate the 5th–95th percentile range around the predicted mean. The suggested therapeutic window (25–100 ng/mL) is indicated by the shaded horizontal region.

Three dosing strategies were assessed: OD, BD and TDS. At all three dosing approaches, predicted mean plasma concentrations were broadly within the therapeutic widow for some of the dosing period (Table 6) (Figure 4); however, for F3, a BD or TDS dosing approach resulted in predicted mean plasma concentrations within the therapeutic window for the entire dosing period, for both age groups. With increasing dosing frequency, Cmax increased by 60% for TDS vs. OD dosing for both age groups with consistent AUCs for each age group (Table 6) (Figure 4).

F1 (immediate release) displayed large fluctuations in peak-to-trough concentrations and a Cmax above the therapeutic range, indicating a negative impact on clinical response and tolerability. Subsequently, the need for an extended-release preparation is advised. F3-given BD was chosen as the optimum dosing approach, as it was able to provide consistent drug plasma concentrations, a reduction in the peak-to-trough fluctuations and potential for improved patient compliance.

### 3.6. Developing a Dosing Approach in Children

Based upon our initial predictions, a BD dosing approach was selected, reflecting a more realistic dosing approach for children compared to TDS dosing utilising F3 (Figure 4). Given that the maximum dose incorporated limitations within each mini-tablet (~10 mg), dosing at age groups above 7 years of age would require more than one mini-tablet BD (Figure 4).

We therefore considered an increase in the overall dose from 250 µg/kg, by 100 µg/kg increments to a maximum dose limit of 10 mg within each mini-tablet dose, with age groups of 3–5- and 5–7-year-olds. A key driver for an optimal dosing approach was to identify a dosing regimen resulting in the fewest subjects with trough plasma concentrations below the lower limit of the therapeutic window, 25 ng/mL, while maintaining the drug loading limits within the formulation.

**Figure 5:**
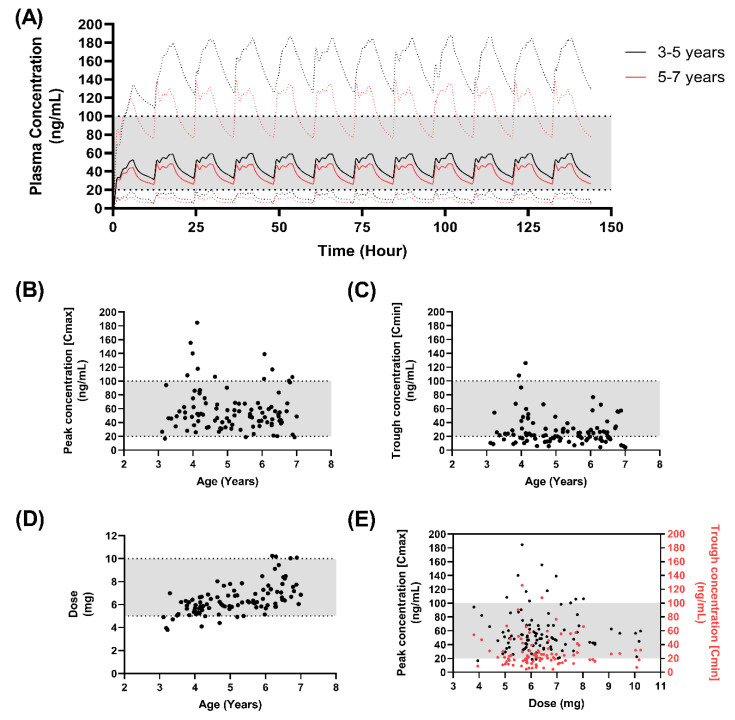
Simulated pharmacokinetics of 350 µg/kg F3 nifedipine mini-tablets in children. (**A**) The predicted mean plasma concentrations of F3 nifedipine, following dosing at 350 µg/kg twice daily in children aged 5–7 years (black line) or 7–11 years (red line) with dashed lines indicating the 5th–95th percentile range around the predicted mean; (**B**) The predicted peak plasma concentrations of F3 nifedipine with age; (**C**) The predicted trough plasma concentrations of F3 nifedipine with age; (**D**) Dose (mg) administered per dosing interval (12 h); (**E**) Peak and trough plasma concentration related to dose (mg) administered per dosing interval (12 h). Shaded horizontal regions indicated the suggested therapeutic window (25–100 ng/mL) (**A**–**E**) or ideal dose range to be incorporated into the mini-tablet (**D**).

A dose of 350 µg/kg resulted in 36% (3–5-year-olds) and 46% (5–7-year-olds) of subjects with trough concentrations below the lower end of the therapeutic window (Table 7) (Figure 5), with peak concentrations broadly within the therapeutic window (Figure 5B) and mean trough concentrations close to the lower limit (3–5-year-olds: 31.76 ng/mL ± 25.9 ng/mL; 5–7-year-olds: 25.6 ng/mL ± 15.6 ng/mL) (Figure 5C).

**Figure 6:**
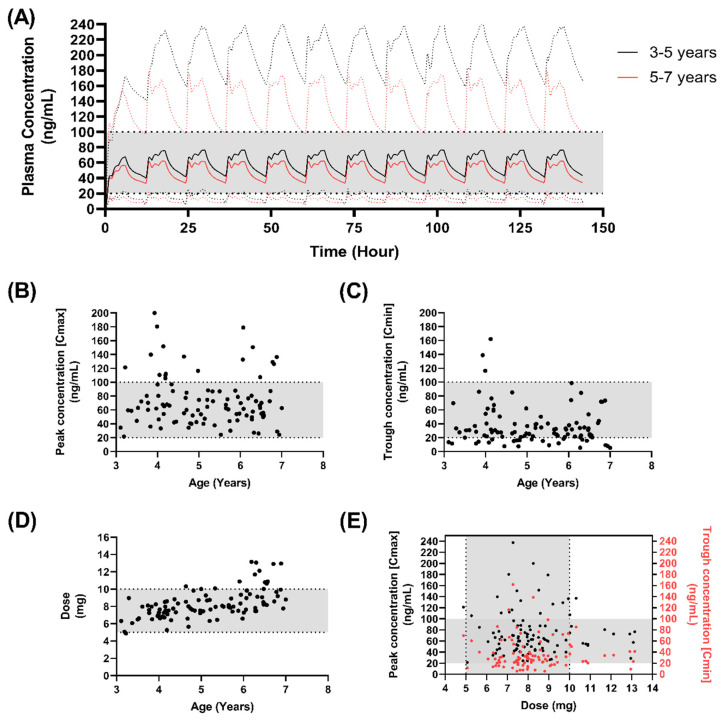
Simulated pharmacokinetics of 450 µg/kg F3 nifedipine mini-tablets in children. (**A**) The predicted mean plasma concentrations of F3 nifedipine, following dosing at 450 µg/kg twice daily in children aged 5–7 years (black line) or 7–11 years (red line) with dashed lines indicating the 5th–95th percentile range around the predicted mean; (**B**) The predicted peak plasma concentrations of F3 nifedipine with age; (**C**) The predicted trough plasma concentrations of F3 nifedipine with age; (**D**) Dose (mg) administered per dosing interval (12 h); (**E**) Peak and trough plasma concentration related to dose (mg) administered per dosing interval (12 h). Shaded horizontal regions indicated the suggested therapeutic window (25–100 ng/mL) (**A**–**E**) or ideal dose range to be incorporated into the mini-tablet (**D**).

However, a dose of 450 µg/kg resulted in the lowest percentage of subjects with trough concentrations below the lower end of the therapeutic window, 23% (3–5-year-olds) and 20% (5–7 year-olds) (Table 7) (Figure 6). Under this dosing strategy, trough concentrations were approximately 30% higher than those for the 350 µg/kg, with the final mean dose being below the 10 mg limit, 7.4 ± 1.1 mg (3–5-year-olds) and 9.04 ± 1.7 (5–7-year-olds).

After an optimised dosing strategy of 450 µg/kg was confirmed, dose banding was used to ensure a maximum of no more than two mini-tablets were dosed at any one time. A strength of 5 mg was selected on the basis of being able to provide enhanced dosing flexibility options (Table 8).

## 4. Conclusions

A key driver for this study was to identify both a clinically informed age-appropriate formulation and a potential dosing strategy within the remits of compliance within paediatrics. The final suggested dosing strategy at 0.45 mg/kg was applicable across the age group of 3–7-year-olds whilst maintaining the limitation on drug loading (<10 mg) to ensure a maximum of no more than two mini-tablets being dosed. Furthermore, this dosing strategy is within the recommended guidelines [63], and has been used in previous studies: (i) children 11.6 ± 5.3 years across a dose of 0.04–0.69 mg/kg [8]; (ii) in a study by Egger, D. W., et al. (2002) [7] who dosed across 0.1–1.2 mg/kg in paediatrics; (iii) in a study by Yiu, V., et al. (2004) [9] with 182 patients aged 0.2–17.9 years with a dosing range of 0.04–0.67 mg/kg per dose. Furthermore, plasma concentration levels observed at a dosing strategy of 0.45 mg/kg are broadly within the range reported by Johnson, C. E., et al. (1991) [64].

However, there is a paucity in nifedipine clinical pharmacokinetics in paediatric age groups, and studies on plasma concentrations in paediatrics would provide clinical context. Despite these drawbacks, this study has, for the first time, provided a pragmatic estimation of a possible dosing approach that could be applied to the use of a novel nifedipine mini-tablet for use in children.

## Figures and Tables

**Table 1:** Kinetic drug release models.

Model	Equation
Zero order	Q_1_ = Q_0_ + K_0_t
First order	Log C_t_ = Log C_0_ − k t/2.303
Higuchi	Q = K_H_ t^1/2^
Korsmeyer–Peppas	Q_t_/Q∞ = Kt^n^

Where Q is the amount of drug released or dissolved, Q_0_ is the initial amount of drug release or dissolved (usually zero), Q_t_/Q∞ is the fraction of drug released at time t, K is the rate constant, and *n* indicates the release mechanism. In situations where the release mechanism is not well known or more than one type of release could be involved, the Peppas model is commonly used where an *n* value < 0.5 = Quasi-Fickian diffusion, *n* equals 0.5 = Fickian diffusion, 0.5 < *n* < 1. 0 = anomalous (non-Fickian transport), *n* equals 1.0 = case II transport and *n* greater than 1 indicates super case II transport drug transport mechanism [35].

**Table 2:** Composition of extended release nifedipine mini-tablets.

Composition	F1 (% *w*/*w*)	F2 (% *w*/*w*)	F3 (% *w*/*w*)	F4 (% *w*/*w*)
Nifedipine	10	10	10	10
Lactose	89.25	59.25	39.25	19.25
HPMC (K4M)	-	30	50	70
Mg stearate	0.5	0.5	0.5	0.5
AEROSIL^®^	0.25	0.25	0.25	0.25

**Table 3:** Kinetic modelling of F1–F4. *n* = release exponent.

Formulation	Zero Order	First Order	Higuchi	Korsmeyer–Peppas	(*n*)
F1 (immediate)	0.645	0.987	0.348	0.331	0.05
F2 (30% HPMC)	0.387	0.717	0.638	0.895	0.15
F3 (50% HPMC)	0.936	0.985	0.993	0.984	0.58
F4 (70% HPMC)	0.932	0.971	0.994	0.990	0.53

**Table 4:** Pharmacokinetic results following validation of the nifedipine adult model.

Study	Dose		Cmax (ng/mL)	tmax (h)	AUC (ng/mL·h)	Mean Cmax Ratio	Mean tmax Ratio	Mean AUC Ratio
Ohashi et al. (1993) [37]	Single (20 mg)	Predicted	245 ± 152	0.6 ± 0.2	501 ± 124	-	-	-
		Observed G1	nr	nr	680 ± 135	-	-	0.74
		Observed G1	nr	nr	809 ± 318	-	-	0.62
		Observed G1	nr	nr	579 ± 191	-	-	0.87
Tateishi et al. (1989) [38]	Single (20 mg)	Predicted	245 ± 152	0.6 ± 0.2	501 ± 124	-	-	-
		Observed	236 ± 70	1 ± 0.9	623 ± 139	1.04	0.6	0.8
Ohashi et al. (1990) [39]	Single (20 mg)	Predicted	238 ± 142	1.1 ± 0.8	357.1 ± 124.9	-	-	-
		Observed	421 ± 177	nr	453.6 ± 176.2	0.57	-	0.79
Smith et al. (1987) [40]	Single (20 mg)	Predicted	101 (62–225)	1 ± 0.5	279 (307–435)	-	-	-
		Observed	131 (97–179)	0.5	266 (322–415)	0.77	2	1.04
	Multiple (10 mg)	Predicted	82 (62–225)	1 ± 0.5	201 (142–235)	-	-	-
		Observed	56 (42–74)	0.5	134 (113–158)	1.46	2	1.5
Wonnerman et al. (2008) [42]	Single OROS (30 mg)	Predicted	21.8 (15.9–42)	5 (3–17)	319 (214–517)	-	-	-
		Observed	17.6 (11.6–30.8)	6 (5–24)	358 (160–906)	1.23	0.83	0.89

Cmax: maximum plasma concentration; tmax: time to maximum plasma concentration; AUC: area under the curve; nr: not reported; -: not calculated. Mean Cmax, tmax and AUC ratio represent the ratio of predicted:observed pharmacokinetic parameter. Data reported as geometric mean ± (standard deviation).

**Table 5:** Pharmacokinetics of mini-tablets in virtual clinical trials of healthy adults.

Formulation	Dose	Cmax (ng/mL)	tmax (h)	AUC (ng/mL·h)
F1	30 mg BD	229 ± 94.2	1.2 ± 0.15	703 ± 425.6
	60 mg OD	466 ± 187	1.2 ± 0.24	1459 ± 876.8
F2	30 mg BD	167.1 ± 70.16	1.23 ± 0.18	617.9 ± 366.8
	60 mg OD	331 ± 133	1.25 ± 0.26	1270.7 ± 751
F3	30 mg BD	76.68 ± 39.5	2.25 ± 1.99	711.6 ± 407
	60 mg OD	126.8 ± 62	5.5 ± 1.34	1432.8 ± 817

F1: No HPMC mini-tablet; F2: 30% HPMC mini-tablet; F3: 50% HPMC mini-tablet; AUC: area under the curve; Cmax: maximum plasma concentration; tmax: time to maximum plasma concentration; Data reported as geometric mean ± (standard deviation).

**Table 6:** Pharmacokinetics of mini-tablets in virtual clinical trials children dosed at 250 µg/kg.

Formulation	Dosing	Age Range (Years)	Cmax (ng/mL)	tmax (h)	AUC (ng/mL·h)
F1	OD	5–7	113	1.2	398.2
		7–11	101.6	1.2	300
	BD	5–7	113	1.6	397.7
		7–11	105.8	1.36	300
	TDS	5–7	113	1.43	398.1
		7–11	107.1	1.38	301
F2	OD	5–7	113	2.2	364
		7–11	101	2.16	274
	BD	5–7	111	2.2	363
		7–11	102	2.16	275
	TDS	5–7	123	2.15	362
		7–11	106	2.13	274
F3	OD	5–7	39	5.3	418
		7–11	31	4.7	314
	BD	5–7	46	3	417
		7–11	37	2.45	314
	TDS	5–7	63	2.4	418
		7–11	49	2.16	313

F1: No HPMC mini-tablet; F2: 30% HPMC mini-tablet; F3: 50% HPMC mini-tablet; AUC: area under the curve; Cmax: maximum plasma concentration; tmax: time to maximum plasma concentration; OD: once daily; BD: twice daily; TDS: three times daily. Data reported as geometric mean (SD omitted for clarity).

**Table 7:** Simulated optimised F3 mini-tablet pharmacokinetics in children.

Dose	Age Range (Years)	Cmax (ng/mL)	Cmin (ng/mL)	tmax (h)	AUC (ng/mL·h)	<0 ng/mL ^a^ (%)	Dose (mg)
350 µg/kg	3–5	62.48 ± 35	31.76 ± 25.9	3.35 ± 2	563.3 ± 376	36	5.75 ± 0.87
	5–7	53.97 ± 25.3	25.6 ± 15.6	2.59 ± 2	458.9 ± 248.3	46	7.03 ± 1.37
450 µg/kg	3–5	80.45 ± 45.8	40.85 ± 33	3.35 ± 2	726 ± 485.2	23	7.4 ± 1.1
	5–7	69.5 ± 32.6	32.8 ± 20.1	2.59 ± 2	590.8 ± 319	20	9.04 ± 1.7

^a^ Percentage of subjects with trough concentration below the lower limited of the therapeutic window. AUC: area under the curve; Cmax: maximum plasma concentration; Cmin: minimum plasma concentration; tmax: time to maximum plasma concentration; Data reported as geometric mean ± (standard deviation).

**Table 8:** Nifedipine dose banding and number of tablets required to fulfil dose requirements.

Dosing Strategy	Average Weight (kg)	Dose (mg)	5 mg Tablets Required
250 μg/kg	3 years: 14 kg 5 years: 18 kg 7 years: 23 kg	3.5 ≈ 5 4.5 ≈ 5 5.8 ≈ 5	1 1 1
350 μg/kg	3 years: 14 kg 5 years: 18 kg 7 years: 23 kg	4.9 ≈ 5 6.3 ≈ 5 8.1 ≈ 10	1 1 2
450 μg/kg	3 years: 14 kg 5 years: 18 kg 7 years: 23 kg	6.3 ≈ 5 8.1 ≈ 10 10.4 ≈ 10	1 2 2

## Data Availability

The data presented in this study is contained within this article.

## Supplementary Materials

The following supporting information can be downloaded at: https://www.mdpi.com/article/10.3390/pharmaceutics15020556/s1, Table S1: Parameters used for the Nifedipine PBPK model (Simcyp V12). Adapted from Chetty, M., et al. (2014) [36]. Table S2: Paediatric demographics used for the Nifedipine PBPK model.
