# Supplementary material for: Virtual Clinical Trials Guided Design of an Age-Appropriate Formulation and Dosing Strategy of Nifedipine for Paediatric Use
Source: Pharmaceutics. 2023 Feb 7;15(2):556. doi: 10.3390/pharmaceutics15020556 (PMC9961156; doi:10.3390/pharmaceutics15020556)
Supplement: Supplementary file 1 [file pharmaceutics-15-00556-s001.zip › pharmaceutics-2097142-supplementary.pdf]

Supplementary Materials

Table S1: Parameters used for the Nifedipine PBPK model (Simcyp V12). Adapted from Chetty, M., et al. (2014) [36].

Parameter (unit)	Value
**Molecular weight (g/mol)**	346.3
**LogP**	2.69
**Compound type**	Monoprotic base
**pKa**	2.82
**Blood:plasma**	0.685
**Fu plasma**	0.039
**Main binding protein**	albumin
**Absorption**	First order absorption for IR nifedipine<br>Mechanistic absorption model (ADAM) for the CR formulation
**fu_{Gut} (fraction of drug unbound in enterocyte)**	1
**Precipitation rate constant (1/h)**	3.67
**Distribution**	Minimal PBPK model
**Vss (L/kg)**	0.57
**Elimination**	
**CYP3A4:oxidation Km (μM)**	10.5
**CYP3A4:oxidation Vmax (pmol/min/mg microsomal protein)**	22
**CYP3A5:oxidation Km (μM)**	31.9
**CYP3A5:oxidation Vmax (pmol/min/mg microsomal protein)**	3.5
**CL_R (renal clearance) (L/h)**	0 (Negligible)

Table S2: Paediatric demographics used for the Nifedipine PBPK model

3-5	
Age	4.08 +/- 0.55 years
Weight	16.19 +/- 2.65 kg
Height	101.63 +/- 6.24 cm
5–7	
Age	6.08 +/- 0.55 years
Weight	20.25 +/- 3.47 kg
Height	115.44 +/- 6.57 cm
7–11	
Age	9.16 +/- 1.10 years
Weight	28.18 +/- 6.18 kg
Height	132.77 +/- 8.54 cm
